# A randomized trial of 7-day doripenem versus 10-day imipenem-cilastatin for ventilator-associated pneumonia

**DOI:** 10.1186/cc11862

**Published:** 2012-11-13

**Authors:** Marin H Kollef, Jean Chastre, Marc Clavel, Marcos I Restrepo, Bart Michiels, Koné Kaniga, Iolanda Cirillo, Holly Kimko, Rebecca Redman

**Affiliations:** 1 Division of Pulmonary and Critical Care Medicine, Washington University School of Medicine, St. Louis, 660 South Euclid Avenue, Campus Box 8052, St. Louis, MO 63110, USA; 2 Service de Réanimation Médicale, Institut de Cardiologie, Groupe Hospitalier Pitié-Salpêtrière, 47-83 boulevard de l'Hôpital, 75651 Paris Cedex 13, France; 3 Centre Hospitalier De Limoges, Hospital Dupuytren 02 Ave, Martin Luther King 87000, Limoges, France; 4 Division of Pulmonary/Critical Care Medicine, University of Texas Health Science Center at San Antonio and Division Pulmonary/Critical Care Medicine, South Texas Veterans Health Care System, 7400 Merton Minter Blvd. (11c6), San Antonio, TX, 78229 USA; 5 Janssen Research and Development, Turnhoutsewig 30, 2340, Beerse, Belguim; 6 Janssen Pharmaceutical Research and Development, 1000 Route 202, Raritan, NJ, 08869 USA

## Abstract

**Introduction:**

The aim of this study was to compare a 7-day course of doripenem to a 10-day course of imipenem-cilastatin for ventilator-associated pneumonia (VAP) due to Gram-negative bacteria.

**Methods:**

This was a prospective, double-blinded, randomized trial comparing a fixed 7-day course of doripenem one gram as a four-hour infusion every eight hours with a fixed 10-day course of imipenem-cilastatin one gram as a one-hour infusion every eight hours (April 2008 through June 2011).

**Results:**

The study was stopped prematurely at the recommendation of the Independent Data Monitoring Committee that was blinded to treatment arm assignment and performed a scheduled review of data which showed signals that were close to the pre-specified stopping limits. The final analyses included 274 randomized patients. The clinical cure rate at the end of therapy (EOT) in the microbiological intent-to-treat (MITT) population was numerically lower for patients in the doripenem arm compared to the imipenem-cilastatin arm (45.6% versus 56.8%; 95% CI, -26.3% to 3.8%). Similarly, the clinical cure rate at EOT was numerically lower for patients with *Pseudomonas aeruginosa *VAP, the most common Gram-negative pathogen, in the doripenem arm compared to the imipenem-cilastatin arm (41.2% versus 60.0%; 95% CI, -57.2 to 19.5). All cause 28-day mortality in the MITT group was numerically greater for patients in the doripenem arm compared to the imipenem-cilastatin arm (21.5% versus 14.8%; 95% CI, -5.0 to 18.5) and for patients with *P. aeruginosa *VAP (35.3% versus 0.0%; 95% CI, 12.6 to 58.0).

**Conclusions:**

Among patients with microbiologically confirmed late-onset VAP, a fixed 7-day course of doripenem was found to have non-significant higher rates of clinical failure and mortality compared to a fixed 10-day course of imipenem-cilastatin. Consideration should be given to treating patients with VAP for more than seven days to optimize clinical outcome.

**Trial Registration:**

ClinicalTrials.gov: NCT00589693

## Introduction

Ventilator-associated pneumonia (VAP) is the most common infection identified in critically ill patients, often due to high risk pathogens, such as *Pseudomonas aeruginosa *and *Acinetobacter baummannii*, and accounts for most of the antibiotic utilization within intensive care units (ICUs) [1,2]. Several guidelines have been published giving recommendations for the treatment of VAP, including the total duration of therapy [3,4]. Unfortunately, the evidence supporting an optimal duration of antibiotic therapy for VAP is limited and primarily based on the results of a single randomized trial [5]. A recent meta-analysis found that for patients with nosocomial pneumonia not due to non-lactose fermenting Gram-negative bacteria (NLFGNB), a short fixed-course (7 or 8 days) of antibiotic therapy may be more appropriate than a prolonged course (10 to 15 days) in terms of reducing the subsequent emergence of antibiotic-resistant pathogens [6]. However, the concern with using short durations of antibiotic therapy is treatment failure and potentially adverse outcomes.

Carbapenems are bactericidal against Gram-negative pathogens that commonly cause VAP, including *P. aeruginosa, A. baumannii *and extended-spectrum beta-lactamase (ESBL) producing enteric bacteria, and are, therefore, recommended for initial empiric therapy for VAP in patients with late-onset disease or individuals with risk factors for infection with multidrug-resistant (MDR) pathogens [3,4,7]. Doripenem 500 mg was shown to be non-inferior to comparator agents in two previous randomized controlled studies in patients with hospital-acquired pneumonia, including VAP, when administered for 7 to 14 days, with the length of therapy guided by the patient's condition and at the discretion of the treating physicians [8,9]. In addition, pharmacokinetic/pharmacodynamic (PK/PD) modeling from data from other studies demonstrated that one gram doses infused over four hours could target pathogens with higher minimum inhibitory concentrations (MICs) and provide a more sustained duration of free drug concentrations above the MIC of most Gram-negative pathogens causing VAP (especially *P. aeruginosa *and *Acinetobacter *spp.) than the 500 mg dose [10,11]. Therefore, we performed an investigation to compare the administration of a higher 1 g dose of doripenem for a fixed 7-day course to a fixed10-day course of imipenem-cilastatin for the treatment of late-onset VAP. The rationale for the use of a 7-day course of doripenem was guided by data from the prior doripenem nosocomial pneumonia registration trials and a previous study demonstrating similar outcomes in patients with VAP treated with 8 and 15 days of antibiotic therapy [5,8,9].

Doripenem is not approved for treatment of nosocomial pneumonia, including VAP, in the United States (US) but is approved for use in adults with these infections in the European Union and other countries outside of the US.

## Materials and methods

### Study design overview

A randomized, double-blind, multicenter study was performed comparing the efficacy and safety of a fixed 7-day regimen of doripenem to a fixed 10-day regimen of imipenem-cilastatin in patients with late-onset VAP, with patients enrolled between 1 April 2008 and 17 May 2011. Ventilated patients were stratified at the time of randomization based on age (≤65 years or >65 years), degree of lung injury as measured by the ratio of the partial pressure of arterial oxygen/fraction of inspired oxygen (PaO2/FiO2 of ≤250 or >250), and geographic region (Western Europe, North America, Australia; Central and South America; or Eastern Europe and Asia). The institutional review board at each site (see Acknowledgements) approved the protocol, and all patients or their authorized representatives provided written informed consent (NCT00589693). (See Additional file 1 for complete Methods section).

### Randomization and treatment regimens

In this double-blinded study patients were randomized (1:1) to receive either a fixed 7-day course of doripenem one gram as a four-hour infusion every eight hours or a fixed 10-day course of imipenem-cilastatin one gram as a one-hour infusion every eight hours. Treatment was randomized with use of a central interactive phone system. Randomization was not stratified by study site. Patients randomized to doripenem treatment received in parallel 7 days of active therapy and 10 days of placebo. Patients randomized to imipenem-cilastatin treatment received in parallel 10 days of active therapy and 7 days of placebo. All patients received active study drug and placebo infusions on Days 1 through 7. Patients randomized to imipenem-cilastatin continued to receive active study drug on Days 8, 9 and 10 and patients randomized to doripenem received placebo. A switch to oral antibacterial therapy was not allowed. Adjunctive therapy was allowed at the discretion of the treating physician with vancomycin (1 gram every 12 hours) or linezolid (600 mg every 12 hours) directed at methicillin-resistant *Staphylococcus aureus *(MRSA) and amikacin (15 mg/kg once daily) for patients at risk for infection with a carbapenem-resistant Gram-negative pathogen.

### Outcomes and follow-up

The intent-to-treat (ITT) population was defined as all patients who received at least one dose of the study drug. The microbiological ITT (MITT) population was the subset of the ITT population who had at least one Gram-negative pathogen identified on bronchoalveolar lavage (BAL) or mini-BAL at a density >10^4 ^CFU/mL with an imipenem MIC <8 μg/mL. Patients were included in the MITT population if they had a second pathogen isolated from BAL/mini-BAL at a density >10^4 ^CFU/mL with an imipenem MIC >8 μg/mL. This was allowed to optimize enrollment of patients with eligible Gram-negative pathogens and to allow inclusion of co-infection with MRSA. However, patients who only grew pneumonia pathogens with imipenem MICs >8 μg/mL, such as MRSA or *Stenotrophomonas maltophilia*, were excluded from the MITT population.

Clinical assessments were performed at baseline and at the end of therapy (EOT), defined as Day 10 for both groups, or within 24 hours after the last dose of blinded study drug therapy if discontinued early. Laboratory assessments were performed at baseline, Day 7 and EOT. Follow-up assessments were conducted 7 to 14 days and 28 to 35 days after EOT. The primary endpoint of this study was clinical cure at EOT (Day 10) in the MITT population. Secondary endpoints included 28-day all- cause mortality in the MITT populations and clinical cure in the subgroup having *P. aeruginosa *identified as a qualifying pathogen.

Clinical cure was defined as improvement or lack of progression of baseline radiographic findings at EOT and resolution of signs and symptoms of pneumonia at follow-up. Failure was defined as persistence or progression of signs and symptoms or progression of radiological signs of pneumonia at EOT; termination of study medications due to "lack of efficacy"; administration of any systemically absorbed or aerosolized antibiotic for any reason; death from any cause; an indeterminate response; or relapsed infection at follow-up after termination of study medications. Adverse events (AEs) including mortality, vital signs and laboratory parameters were also evaluated.

### Statistical analysis

The initial sample size calculation was based on assumptions from a previous Phase 3 doripenem pneumonia study conducted in patients with VAP [9). Assuming a clinical cure rate of 60% in both treatment arms and using a non-inferiority margin of 15% and a one-tailed 2.5% significance level, a sample size of 168 per treatment arm would have a power of 80% to establish non-inferiority. If one further assumed that only 70% of the patients would qualify for inclusion in the MITT analysis set, then the sample size required would be 240 per treatment arm for a total of 480 patients. Categorical data were expressed as frequency distributions and the difference between groups was assessed by using the normal approximation to the difference between two binomial proportions. All confidence intervals were two-tailed and a *P*-value <0.05 represented statistical significance. No correction for multiple comparisons was implemented. The *P*-values in the secondary and subgroup analyses are nominal in nature, and not inferential.

### Independent Data Monitoring Committee

**Figure 1:**
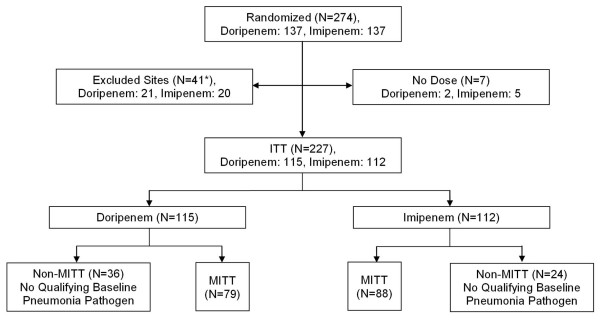
**Patients enrolled and analyzed**. ITT, intention-to-treat; MITT, Microbiological intention-to-treat. *Prior to study termination the Marketing Authorization Holder for the study identified five study sites (three in Guatemala, one in Germany, one in the United States), following independent internal reviews and re-monitoring by a contract research organization (CRO), that were found not to have adhered to the study protocols, or the study logs could not verify protocol adherence, and thus their data were excluded from the primary analyses.

An Independent Data Monitoring Committee (IDMC) was established to evaluate data related to efficacy and safety at predefined time points (see on-line supplement for IDMC statistical monitoring guidelines). At their last meeting, the IDMC reviewed available data from approximately half the total number of patients targeted for enrollment and recommended that the enrollment be terminated because of inferior efficacy and higher mortality in one of the treatment arms. Therefore, the analyses were based on data from the 274 subjects who had been randomized into the study at the time enrollment was terminated. In addition, five sites (three in Guatemala, one in Germany, one in the United States) that enrolled a total of 41 patients were deemed to be non-compliant with good clinical practices (GCP) prior to database lock and were excluded from the primary analyses of efficacy and safety (Figure 1). However, to assess the robustness of the primary efficacy and safety conclusions, sensitivity analyses were performed by including patients from these five sites. These sensitivity analyses support the primary efficacy and safety conclusions.

## Results

### Patient disposition and characteristics

There were 274 randomized patients prior to stopping the study. In addition to the 41 patients from the GCP non-compliant sites, 7 patients were excluded who never received the study drug (1 patient was excluded for meeting both of these criteria). The ITT group comprised 227 patients (doripenem, *n *= 115; imipenem-cilastatin, *n *= 112) and the MITT group comprised 167 patients (doripenem, *n *= 79; imipenem-cilastatin, *n *= 88) (Figure 1). Patient baseline characteristics were generally balanced between treatment groups for the ITT and MITT populations although there were some differences between treatment groups suggesting subjects in the doripenem arm may have more severe illness. The majority of patients were male, white, <65 years of age (mean of 54.1 years in the MITT population), had an Acute Physiology and Chronic Health Evaluation (APACHE) II score >15, a clinical pulmonary infection score (CPIS) ≥6, P_a_O_2_/F_i_O_2 _<250, a creatinine clearance >50 ml/min, received >72 hours of prior antibiotic therapy, and enrolled from sites in Europe (Tables 1 and 2). The most common reasons patients in the MITT population were admitted to the hospital were for surgery (38.9%), including neurologic surgery (17.4%), a neurologic event (20.4%) and trauma (18.6%). A qualifying Gram-negative pathogen was isolated from 89.2% of patients in the MITT population and more than half of these patients had a second pathogen isolated from the baseline BAL/mini-BAL at a density >10^4 ^CFU/mL.

**Table 1:** Baseline demographics - ITT analysis set

	Doripenem	Imipenem	Total
	(*N *= 115)	(*N *= 112)	(*N *= 227)
**Sex, n (%) **			
N	115	112	227
Male	72 (62.6)	75 (67.0)	147 (64.8)
Female	43 (37.4)	37 (33.0)	80 (35.2)
**Weight (kg) **			
N	115	112	227
Mean (SD)	75.6 (16.95)	79.8 (19.07)	77.7 (18.11)
Median	74.0	77.5	75.0
Range	(45;150)	(47; 170)	(45; 170)
**Height (cm) **			
N	114	110	224
Mean (SD)	169.5 (10.66)	171.3 (9.20)	170.3 (9.99)
Median	170.0	170.0	170.0
Range	(140; 193)	(150; 200)	(140; 200)
**Age (Years) **			
N	115	112	227
Mean (SD)	57.5 (16.53)	54.6 (18.46)	56.1 (17.53)
Median	58.0	58.0	58.0
Range	(19; 89)	(18; 88)	(18; 89)
**Race, n (%) **			
N	115	112	227
White	96 (83.5)	97 (86.6)	193 (85.0)
Black or African American	6 (5.2)	6 (5.4)	12 (5.3)
Asian	4 (3.5)	1 (0.9)	5 (2.2)
Other	9 (7.8)	8 (7.1)	17 (7.5)
**Region, n (%) **			
N	115	112	227
Central and South America	24 (20.9)	24 (21.4)	48 (21.1)
Eastern Europe and Asia	34 (29.6)	33 (29.5)	67 (29.5)
Western Europe, North America, Australia	57 (49.6)	55 (49.1)	112 (49.3)
**APACHE II score, n (%) **			
N	115	112	227
≤15	48 (41.7)	49 (43.8)	97 (42.7)
16 to 19	30 (26.1)	34 (30.4)	64 (28.2)
≥20	37 (32.2)	29 (25.9)	66 (29.1)
**CPIS, n (%) **			
N	115	112	227
Missing	3 (2.6)	2 (1.8)	5 (2.2)
<6	8 (7.0)	5 (4.5)	13 (5.7)
6 to 7	64 (55.7)	64 (57.1)	128 (56.4)
8 to 9	30 (26.1)	34 (30.4)	64 (28.2)
>9	10 (8.7)	7 (6.3)	17 (7.5)
**SOFA Score**			
N	57	58	115
Mean (SD)	6.0 (2.70)	5.5 (2.39)	5.8 (2.55)
Median	6.0	5.0	5.0
Range	(0; 14)	(2; 12)	(0; 14)
**Charlson Comorbidity Index**			
N	114	112	226
Mean (SD)	3.0 (2.72)	2.8 (2.45)	2.9 (2.59)
Median	3.0	3.0	3.0
Range	(0; 14)	(0; 9)	(0; 14)
**PaO_2_/FiO_2_, n (%)**			
N	115	112	227
≤250	67 (58.3)	61 (54.5)	128 (56.4)
>250	48 (41.7)	51 (45.5)	99 (43.6)
**Bacteremia, n (%)**			
N	115	112	227
No	109 (94.8)	107 (95.5)	216 (95.2)
Yes	6 (5.2)	5 (4.5)	11 (4.8)
**Creatinine clearance, n (%) **			
N	115	112	227
Supra normal (≥150 ml/min)	23 (20.0)	34 (30.4)	57 (25.1)
Normal (≥80 to <150 ml/min)	46 (40.0)	44 (39.3)	90 (39.6)
Mild renal failure (>50 to <80 ml/min)	33 (28.7)	26 (23.2)	59 (26.0)
Moderate renal failure (>30 to ≤50 ml/min)	7 (6.1)	4 (3.6)	11 (4.8)
Severe renal failure (≤30 ml/min)	6 (5.2)	4 (3.6)	10 (4.4)
**Failed antibiotic treatment for VAP, n (%) **			
N	69	78	147
No	59 (85.5)	67 (85.9)	126 (85.7)
Yes	10 (14.5)	11 (14.1)	21 (14.3)
**Prior antibacterial therapy usage (hours), n (%)**			
N	115	112	227
<24	28 (24.3)	35 (31.3)	63 (27.8)
≥24 to <48	10 (8.7)	10 (8.9)	20 (8.8)
≥48 to ≤72	9 (7.8)	5 (4.5)	14 (6.2)
>72	68 (59.1)	62 (55.4)	130 (57.3)
**Adjunctive therapy, n (%)**			
N	115	112	227
No	75 (65.2)	79 (70.5)	154 (67.8)
Yes			
≤72 Hrs	32 (27.8)	25 (22.3)	57 (25.1)
>72 Hrs	8 (7.0)	8 (7.1)	16 (7.0)
**Adjunctive aminoglycoside, n (%)**			
N	40	33	73
No	19 (47.5)	20 (60.6)	39 (53.4)
Yes			
≤72 Hrs	20 (50.0)	12 (36.4)	32 (43.8)
>72 Hrs	1 (2.5)	1 (3.0)	2 (2.7)
**Adjunctive vancomycin/linezolid, n (%)**			
N	40	33	73
No	14 (35.0)	9 (27.3)	23 (31.5)
Yes			
≤72 hrs	20 (50.0)	18 (54.5)	38 (52.1)
>72 hrs	6 (15.0)	6 (18.2)	12 (16.4)

APACHE, Acute Physiology and Chronic Health Evaluation; CPIS, Clinical Pulmonary Infection Score; ITT, intention-to-treat; SOFA, Sequential Organ Failure Assessment

**Table 2:** Baseline demographics - MITT analysis set

	Doripenem	Imipenem	Total
	(*N *= 79)	(*N *= 88)	(*N *= 167)
**Sex, n (%) **			
N	79	88	167
Male	48 (60.8)	61 (69.3)	109 (65.3)
Female	31 (39.2)	27 (30.7)	58 (34.7)
**Weight (kg) **			
N	79	88	167
Mean (SD)	75.5 (17.85)	78.7 (15.66)	77.2 (16.75)
Median	75.0	78.0	76.0
Range	(45; 150)	(47; 143)	(45; 150)
**Height (cm) **			
N	79	87	166
Mean (SD)	170.8 (9.76)	171.7 (8.73)	171.3 (9.22)
Median	170.0	170.0	170.0
Range	(148; 193)	(150; 190)	(148; 193)
**Age (years) **			
N	79	88	167
Mean (SD)	54.9 (16.10)	53.4 (18.94)	54.1 (17.62)
Median	56.0	57.0	57.0
Range	(19; 89)	(18; 88)	(18; 89)
**Race, n (%) **			
N	79	88	167
White	65 (82.3)	75 (85.2)	140 (83.8)
Black or African American	5 (6.3)	6 (6.8)	11 (6.6)
Asian	1 (1.3)	1 (1.1)	2 (1.2)
Other	8 (10.1)	6 (6.8)	14 (8.4)
**Region, n (%) **			
N	79	88	167
Central and South America	20 (25.3)	19 (21.6)	39 (23.4)
Eastern Europe and Asia	24 (30.4)	27 (30.7)	51 (30.5)
Western Europe, North America, Australia	35 (44.3)	42 (47.7)	77 (46.1)
**APACHE II score group, n (%) **			
N	79	88	167
≤15	34 (43.0)	42 (47.7)	76 (45.5)
16 to 19	24 (30.4)	21 (23.9)	45 (26.9)
≥20	21 (26.6)	25 (28.4)	46 (27.5)
**CPIS, n (%) **			
N	79	88	167
Missing	2 (2.5)	1 (1.1)	3 (1.8)
<6	4 (5.1)	2 (2.3)	6 (3.6)
6 to 7	43 (54.4)	50 (56.8)	93 (55.7)
8 to 9	23 (29.1)	29 (33.0)	52 (31.1)
>9	7 (8.9)	6 (6.8)	13 (7.8)
**SOFA score**			
N	41	46	87
Mean (SD)	5.7 (2.53)	5.2 (2.24)	5.4 (2.38)
Median	6.0	5.0	5.0
Range	(0; 11)	(2; 12)	(0; 12)
**Charlson Comorbidity Index**			
N	79	88	167
Mean (SD)	2.4 (2.08)	2.8 (2.48)	2.6 (2.30)
Median	3.0	2.0	3.0
Range	(0; 9)	(0; 9)	(0; 9)
**PaO_2_/FiO_2_, n (%)**			
N	79	88	167
≤250	50 (63.3)	51 (58.0)	101 (60.5)
>250	29 (36.7)	37 (42.0)	66 (39.5)
**Bacteremia, n (%)**			
N	79	88	167
No	73 (92.4)	84 (95.5)	157 (94.0)
Yes	6 (7.6)	4 (4.5)	10 (6.0)
**Creatinine Clearance, n (%)**			
N	79	88	167
Supra normal (≥150 ml/min)	18 (22.8)	28 (31.8)	46 (27.5)
Normal (≥80 to <150 ml/min)	31 (39.2)	37 (42.0)	68 (40.7)
Mild renal failure (>50 to <80 ml/min)	23 (29.1)	18 (20.5)	41 (24.6)
Moderate renal failure (>30 to ≤50 ml/min)	5 (6.3)	2 (2.3)	7 (4.2)
Severe renal failure (≤30 ml/min)	2 (2.5)	3 (3.4)	5 (3.0)
**Failed antibiotic treatment, n (%)**			
N	55	68	123
No	45 (81.8)	58 (85.3)	103 (83.7)
Yes	10 (18.2)	10 (14.7)	20 (16.3)
**Prior antibacterial therapy usage (hours), n (%)**			
N	79	88	167
<24	19 (24.1)	29 (33.0)	48 (28.7)
≥24 to <48	8 (10.1)	8 (9.1)	16 (9.6)
≥48 to ≤72	6 (7.6)	3 (3.4)	9 (5.4)
>72	46 (58.2)	48 (54.5)	94 (56.3)
**Adjunctive therapy, n (%)**			
N	79	88	167
No	49 (62.0)	68 (77.3)	117 (70.1)
Yes			
≤72 Hrs	23 (29.1)	15 (17.0)	38 (22.8)
>72 Hrs	7 (8.9)	5 (5.7)	12 (7.2)
**Adjunctive aminoglycoside, n (%)**			
N	30	20	50
No	15 (50.0)	11 (55.0)	26 (52.0)
Yes			
≤72 hrs	14 (46.7)	8 (40.0)	22 (44.0)
>72 hrs	1 (3.3)	1 (5.0)	2 (4.0)
**Adjunctive vancomycin/linezolid, n (%)**			
N	30	20	50
No	9 (30.0)	6 (30.0)	15 (30.0)
Yes			
≤72 hrs	16 (53.3)	11 (55.0)	27 (54.0)
>72 hrs	5 (16.7)	3 (15.0)	8 (16.0)

APACHE, Acute Physiology and Chronic Health Evaluation; CPIS, Clinical Pulmonary Infection Score; MITT, microbiological intention-to -treat; SOFA, Sequential Organ Failure Assessment

The median duration of study drug therapy (including placebo) was 9.7 days for each treatment arm in the MITT population. The median duration of active study drug therapy (excluding placebo) was 7.0 days in the doripenem arm and 10.0 days in the imipenem-cilastatin arm for the MITT population. Similar numbers of patients received empiric adjunctive therapy with an aminoglycoside or an anti-MRSA drug and less than 10% in both the ITT and MITT groups continued adjunctive antibiotics beyond 72 hours after a carbapenem-resistant pathogen (defined as imipenem MIC >8 μg/mL) was isolated (Tables 1 and 2).

### Clinical and microbiologic response

**Figure 2:**
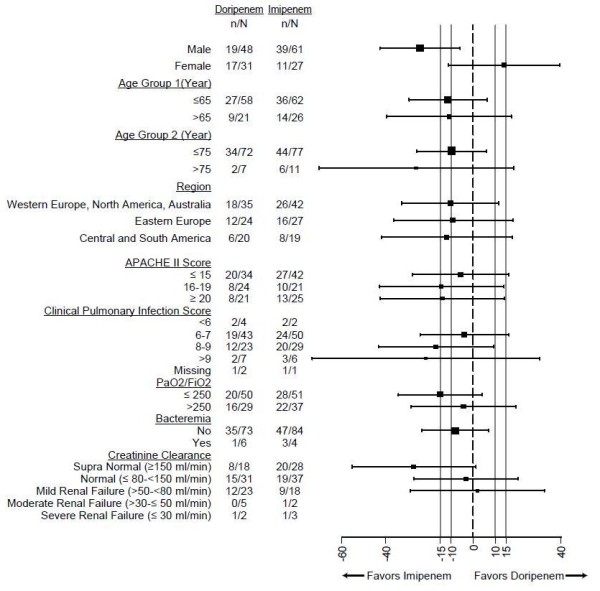
**Clinical cure rates at end of treatment by subgroup with 95% confidence intervals**.

The clinical cure rate at the EOT visit in patients in the MITT group randomized to doripenem was lower than the clinical cure rate in patients randomized to imipenem-cilastatin (45.6% versus 56.8%; 95% CI, -26.3% to 3.8%). Thus, non-inferiority of a fixed 7-day treatment regimen with doripenem compared to a fixed 10-day treatment regimen of imipenem-cilastatin was not demonstrated at the 15% margin. Response differences of 10% to 15% favoring imipenem-cilastatin remained present in most subgroups (Figure 2), especially among male patients and those with supra-normal creatinine clearance. However, the larger differences in cure rates in patients with supra-normal creatinine clearance appear to be driven by the unusually high cure rates among subjects in the imipenem-cilastatin arm with creatinine clearance >150 ml/min (71.4%) compared to cure rates among those with creatinine clearance >80 to <150 ml/min (51.4%) and >50 to <80 ml/min (50.0%).

**Figure 3:**
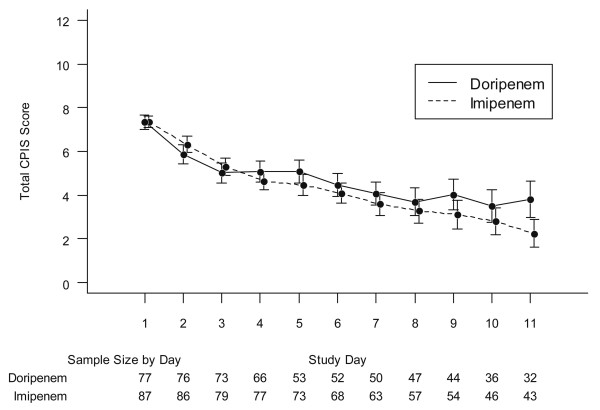
**Mean Clinical Pulmonary Infection Scores (CPIS) for the MITT treatment groups during antibiotic therapy**. Error bars displayed are based on the 95% confidence interval around the means. As there is significant dropout over time, as can be seen by the available sample sizes at the bottom of the figure, the results have to be interpreted with caution. Nevertheless, the curves suggest that the patients' improvement is similar for the two treatment arms up to Day 8 (the last day of active doripenem treatment), where after the decreasing trend is continued for the subjects in the Imipenem-cilastatin arm (who receive active treatment up to Day 11), but remains stable for subjects in the doripenem arm (who receive only placebo from Day 9 up to Day 11).

The mean CPIS values for MITT patients in both treatment arms for study Days 1 through 11 is shown in Figure 3. CPIS scores were similar for patients in the doripenem arm and the imipenem-cilastatin arm for the first eight days of the study. However, the CPIS scores separated after Day 8 with the doripenem arm scores remaining stable while the imipenem-cilastatin arm scores continued to decrease.

The distribution of qualifying Gram-negative pathogens that were pre-defined as being of specific interest (*P. aeruginosa, Acinetobacter *spp., and Enterobacteriaceae) is shown in Table 3. A larger proportion of patients in the doripenem arm than the imipenem-cilastatin arm had pneumonia due to *P. aeruginosa *(21.5% versus 11.4%) and *Acinetobacter *spp. (19.0% versus 11.4%). The clinical cure rate for the *P. aeruginosa *subgroup at EOT was numerically lower for subjects in the doripenem arm compared to the imipenem-cilastatin arm (41.2% (7/17) versus 60.0% (6/10); 95% CI, -57.2% to 19.5%). Cure rates were also lower for patients in the doripenem arm infected with *Acinetobacter *spp. (40.0% (6/15) versus 50.0% (5/10); 95% CI: -49.7% to 29.7%) and Enterobacteriaceae (53.5% (23/43) versus 59.2% (29/49); 95% CI: -26.0% to 14.6%). Table 4 shows that the baseline characteristics for the *P. aeruginosa *subgroup were similar between treatment arms.

**Table 3:** Distribution of baseline qualifying Gram-negative pathogens

	Doripenem	Imipenem	Total
	(*N *= 79)	(*N *= 88)	(*N *= 167)
	n (%)	n (%)	n (%)
**Specific Gram-negative Pathogens**	65 (82.3)	62 (70.5)	127 (76.0)
*Pseudomonas aeruginosa*	17 (21.5)	10 (11.4)	27 (16.2)
Monomicrobial*	*8 (*10.1)	6 (6.8)	14 (8.4)
Polymicrobial**	9 (11.4)	4 (4.5)	13 (7.8)
*Acinetobacter *spp.	15 (19.0)	10 (11.4)	25 (15.0)
Monomicrobial*	3 (3.8)	4 (4.5)	7 (4.2)
Polymicrobial**	12 (15.2)	6 (6.8)	18 (10.8)
*Enterobacteriaceae*	43 (54.4)	49 (55.7)	92 (55.1)
Monomicrobial*	14 (17.7)	14 (15.9)	28 (16.8)
*Citrobacter koseri*	1 (1.3)	0	1 (0.6)
*Enterobacter aerogenes*	0	1 (1.1)	1 (0.6)
*Enterobacter cloacae*	4 (5.1)	3 (3.4)	7 (4.2)
*Escherichia coli*	1 (1.3)	2 (2.3)	3 (1.8)
*Klebsiella pneumoniae*	5 (6.3)	4 (4.5)	9 (5.4)
*Pantoea agglomerans*	1 (1.3)	0	1 (0.6)
*Proteus mirabilis*	1 (1.3)	2 (2.3)	3 (1.8)
*Serratia marcescens*	1 (1.3)	2 (2.3)	3 (1.8)
Polymicrobial**	29 (36.7)	35 (39.8)	64 (38.3)
*Citrobacter freundii*	0	3 (3.4)	3 (1.8)
*Enterobacter aerogenes*	0	2 (2.3)	2 (1.2)
*Enterobacter asburiae*	1 (1.3)	0	1 (0.6)
*Enterobacter cloacae*	4 (5.1)	2 (2.3)	6 (3.6)
*Enterobacter *spp. (Not Speciated)	1 (1.3)	0	1 (0.6)
*Escherichia coli*	5 (6.3)	12 (13.6)	17 (10.2)
*Klebsiella oxytoca*	1 (1.3)	1 (1.1)	2 (1.2)
*Klebsiella pneumoniae*	14 (17.7)	16 (18.2)	30 (18.0)
*Proteus mirabilis*	3 (3.8)	3 (3.4)	6 (3.6)
*Proteus vulgaris*	1 (1.3)	1 (1.1)	2 (1.2)
*Providencia *spp. (Not Speciated)	0	1 (1.1)	1 (0.6)
*Serratia marcescens*	3 (3.8)	3 (3.4)	6 (3.6)
**Pneumonia with MRS**	7 (8.9)	4 (4.5)	11 (6.6)
*Pseudomonas aeruginosa*	2 (2.5)	1 (1.1)	3 (1.8)
Polymicrobial**	2 (2.5)	1 (1.1)	3 (1.8)
*Acinetobacter *spp.	2 (2.5)	1 (1.1)	3 (1.8)
Polymicrobial**	2 (2.5)	1 (1.1)	3 (1.8)
*Enterobacteriaceae*	4 (5.1)	2 (2.3)	6 (3.6)
Polymicrobial**	4 (5.1)	2 (2.3)	6 (3.6)

*: Monomicrobial pneumonia means a single qualifying pneumonia pathogen was isolated and no other pneumonia pathogen was isolated. **: Polymicrobial pneumonia means at least one qualifying pneumonia pathogen was isolated and at least one other pneumonia pathogen was isolated. MRS, methicillin-resistant *Staphylococcus*

**Table 4:** Baseline clinical characteristics - *P*.

	Doripenem	Imipenem	Total
	(*N *= 17)	(*N *= 10)	(*N *= 27)
**Sex, n (%) **			
N	17	10	27
Male	13 (76.5)	7 (70.0)	20 (74.1)
Female	4 (23.5)	3 (30.0)	7 (25.9)
**Weight (kg)**			
N	17	10	27
Mean (SD)	74.0 (17.85)	68.3 (12.12)	71.9 (15.96)
Median	75.0	64.0	72.7
Range	(45; 110)	(52; 90)	(45; 110)
**Height (cm)**			
N	17	10	27
Mean (SD)	172.0 (10.11)	170.8 (7.74)	171.6 (9.17)
Median	170.0	171.0	170.0
Range	(156; 190)	(159; 180)	(156; 190)
**Age (Years)**			
N	17	10	27
Mean (SD)	57.5 (15.58)	50.9 (20.32)	55.1 (17.40)
Median	57.0	53.5	57.0
Range	(33; 89)	(25; 77)	(25; 89)
**Race, n (%)**			
N	17	10	27
White	16 (94.1)	7 (70.0)	23 (85.2)
Black or African American	1 (5.9)	2 (20.0)	3 (11.1)
Asian	0	1 (10.0)	1 (3.7)
**Region, n (%) **			
N	17	10	27
Central and South America	5 (29.4)	3 (30.0)	8 (29.6)
Eastern Europe and Asia	5 (29.4)	4 (40.0)	9 (33.3)
Western Europe, North America, Australia	7 (41.2)	3 (30.0)	10 (37.0)
**APACHE II score group, n (%) **			
N	17	10	27
≤15	6 (35.3)	3 (30.0)	9 (33.3)
16 to 19	5 (29.4)	3 (30.0)	8 (29.6)
≥20	6 (35.3)	4 (40.0)	10 (37.0)
**CPIS, n (%)**			
N	17	10	27
<6	0	1 (10.0)	1 (3.7)
6 to 7	10 (58.8)	8 (80.0)	18 (66.7)
8 to 9	5 (29.4)	1 (10.0)	6 (22.2)
>9	2 (11.8)	0	2 (7.4)
**SOFA score**			
N	10	4	14
Mean (SD)	5.4 (3.10)	4.0 (1.41)	5.0 (2.75)
Median	4.0	4.5	4.0
Range	(1; 10)	(2; 5)	(1; 10)
**Charlson Comorbidity Index**			
N	17	10	27
Mean (SD)	3.0 (2.26)	2.5 (1.96)	2.8 (2.13)
Median	3.0	3.0	3.0
Range	(0; 9)	(0; 5)	(0; 9)
**PaO_2_/FiO_2_, n (%) **			
N	17	10	27
≤250	10 (58.8)	5 (50.0)	15 (55.6)
>250	7 (41.2)	5 (50.0)	12 (44.4)
**Bacteremia, n (%) **			
N	17	10	27
No	16 (94.1)	10 (100)	26 (96.3)
Yes	1 (5.9)	0	1 (3.7)
**Creatinine clearance, n (%) **			
N	17	10	27
Supra normal(≥150 ml/min)	5 (29.4)	4 (40.0)	9 (33.3)
Normal (≥80 to <150 ml/min)	5 (29.4)	2 (20.0)	7 (25.9)
Mild renal failure (>50 to <80 ml/min)	4 (23.5)	3 (30.0)	7 (25.9)
Moderate renal failure (>30 to ≤50 ml/min)	2 (11.8)	1 (10.0)	3 (11.1)
Severe renal failure (≤30 ml/min)	1 (5.9)	0	1 (3.7)
**Failed antibiotic treatment, n (%) **			
N	11	8	19
No	8 (72.7)	6 (75.0)	14 (73.7)
Yes	3 (27.3)	2 (25.0)	5 (26.3)
**Prior antibacterial therapy usage (hours), n (%) **			
N	17	10	27
<24	4 (23.5)	0	4 (14.8)
≥24 to <48	2 (11.8)	1 (10.0)	3 (11.1)
≥48 to ≤72	1 (5.9)	0	1 (3.7)
>72	10 (58.8)	9 (90.0)	19 (70.4)
**Adjunctive therapy, n (%)**			
N	17	10	27
No	7 (41.2)	6 (60.0)	13 (48.1)
Yes			
≤72 hrs	8 (47.1)	3 (30.0)	11 (40.7)
>72 hrs	2 (11.8)	1 (10.0)	3 (11.1)
**Adjunctive aminoglycoside, n (%)**			
N	10	4	14
No	4 (40.0)	2 (50.0)	6 (42.9)
Yes			
≤72 hrs	5 (50.0)	2 (50.0)	7 (50.0)
>72 hrs	1 (10.0)	0	1 (7.1)
**Adjunctive vancomycin/linezolid, n (%)**			
N	10	4	14
No	5 (50.0)	1 (25.0)	6 (42.9)
Yes			
≤72 hrs	4 (40.0)	2 (50.0)	6 (42.9)
>72 hrs	1 (10.0)	1 (25.0)	2 (14.3)

*aeruginosa *analysis set APACHE, Acute Physiology and Chronic Health Evaluation; CPIS, Clinical Pulmonary Infection Score; SOFA, Sequential Organ Failure Assessment

The number of patients with pathogens at each MIC was too small to draw definitive conclusions regarding clinical cure rate by infecting pathogen MIC; however, for the NLFGNB *P. aeruginosa *and *A. baumannii*, cure rates and mortality for patients in either treatment arm did not appear to increase with increasing MIC of the study drug received suggesting conditions other than MIC played a role in outcome (see Additional file 1, Table S1).

### Safety

In the ITT population, the most frequently reported adverse events in both treatment groups were anemia (21.7% doripenem, 22.3% imipenem-cilastatin); urinary tract infection (13.0% doripenem, 14.3% imipenem-cilastatin); decubitus ulcer (12.2% doripenem, 9.8% imipenem-cilastatin); hypokalemia (10.4% doripenem, 10.7% imipenem-cilastatin); diarrhea (9.6% doripenem, 11.6% imipenem-cilastatin); and hypotension (9.6% doripenem, 8.9% imipenem-cilastatin). Clinically important adverse events (all-causality) are shown in Additional file 1. Laboratory results were also comparable between the study arms (see Additional file 1, Table S2).

**Figure 4:**
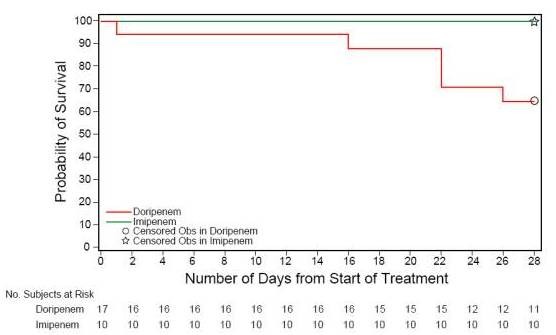
**Kaplan-Meier curves for the *P***. *aeruginosa *subgroup. (*P *= 0.040, Log-Rank Test).

All-cause 28-day mortality in the MITT group was numerically higher for patients in the doripenem arm compared to the imipenem-cilastatin arm (21.5% (17/79) doripenem, 14.8% (13/88) imipenem-cilastatin; 95% CI, -5.0% to 18.5%) and greater for patients with *P. aeruginosa *VAP (35.3% (6/17) versus 0.0% (0/10); 95% CI, 12.6% to 58.0%). Similar trends but smaller mortality differences were demonstrated for the sensitivity analyses for the MITT group (20.7% (19/92) doripenem, 16.7% (17/102) imipenem-cilastatin; 95% CI, -7.0% to 15.0%). Kaplan-Meier estimates are presented graphically for 28-day all-cause mortality for the ITT and MITT populations (see Additional file 1). Figure 4 demonstrates that the Kaplan-Meier curves for the *P. aeruginosa *subgroup are statistically, significantly different over the treatment arms (nominal *P*-value = 0.040) with an increased separation after completion of study drug administration. Notably, no patient in the imipenem arm with *P. aeruginosa *VAP died. In contrast, mortality rates for patients with *Acinetobacter *spp. VAP were lower for patients treated with doripenem (13.3% (2/15) versus 30.0% (3/10); 95% CI: -49.9% to 16.5%).

### Pharmacokinetics

The concentration data collected from 43 subjects treated with doripenem (between study Days 2 and 3) were within the range of historical data in previously studied critically ill patients administered doripenem 1 g for a four-hour infusion. This data were utilized in a population PK/PD analysis along with data from subjects with VAP from previously conducted studies (manuscript under preparation). Higher volumes of distribution were observed in this study population, likely attributable to high peripheral fluid volumes and the VAP disease state. Despite this, doripenem levels were maintained at levels sufficient to target pathogens isolated from subjects in this study.

## Discussion

The main reasons to consider the use of shorter courses of antibiotic therapy for VAP are to minimize antibiotic-related complications and to prevent the emergence of antibiotic resistance. However, we demonstrated that among patients with microbiologically confirmed VAP, a fixed 7-day course of doripenem (one gram as a four-hour infusion every eight hours) had non-significant higher rates of clinical failure and mortality compared to a fixed 10-day course of imipenem-cilastatin (one gram as a one-hour infusion every eight hours). Moreover, patients with VAP attributed to *P. aeruginosa *had a statistically greater risk of 28-day all-cause mortality when treated with doripenem compared to imipenem-cilastatin with an increased separation in the survival curves after completion of study drug administration. This occurred despite the use of prolonged infusions of doripenem aimed at optimizing antibiotic concentration target attainment above the MIC of bacterial pathogens during the dosing interval suggesting that the shorter course of doripenem administration played a role in this survival difference [12,13].

Our findings are in contrast to some earlier studies suggesting that shorter courses of antibiotic therapy for VAP are safe and efficacious compared to longer treatment courses. Ibrahim *et al. *showed that implementation of a clinical guideline for the treatment of VAP was associated with greater administration of appropriate initial antimicrobial treatment [14]. The duration of antimicrobial treatment was also statistically shorter with use of the guideline (8.6 + 5.1 days versus 14.8 + 8.1 days, *P *<0.001) and second episodes of VAP occurred statistically less often. In a subsequent study the same group of investigators found that an antibiotic discontinuation policy for clinically suspected VAP, overseen by clinical pharmacists who were part of the ICU team, could also significantly reduce the duration of antibiotic therapy compared to therapy determined by the treating physician teams (6.0 + 4.9 days versus 8.0 + 5.6 days, *P *= 0.001) [15]. Secondary outcomes including relapse of VAP, hospital mortality, and lengths of stay were similar between groups, although the number of infections attributed to NLFGNB was small. Several groups have also employed prediction tools like CPIS and the biomarker procalcitonin to successfully reduce the duration of antimicrobial therapy in patients with VAP without adversely influencing patient outcomes [16-18].

However, a number of studies suggest that shorter courses of antibiotic therapy for VAP may potentially be less favorable in some circumstances, especially for treatment of infections attributed to NLFGNB. Chastre *et al. *showed that among patients with VAP, all of whom received appropriate initial empiric antibiotic therapy, comparable clinical effectiveness and outcomes were obtained with 8- and 15-day treatment regimens [5]. Yet, patients with VAP caused by NLFGNB, including *P. aeruginosa*, receiving 8 days of treatment had a higher pulmonary infection recurrence rate compared with those receiving 15 days of treatment (40.6% versus 25.4%; 95% CI, 3.9% to 26.6%). Hedrick *et al. *retrospectively evaluated 154 patients with VAP attributed to NLFGNB where 27 patients were treated with three to eight days (mean 6.4 + 0.3 days) of antibiotics and 127 received nine or more days (mean 17.1 + 0.7 days) of therapy [19]. Although not statistically different, the mortality rate was higher for patients receiving the shorter treatment courses (22% versus 14%; *P *= 0.38). Other investigators have demonstrated that longer courses of antibiotic therapy (10 to 14 days) are typically needed to successfully treat VAP attributed to MDR Gram-negative bacteria, often due to the presence of initial inappropriate antibiotic treatment [20,21].

Several potential explanations may have accounted for our findings. The importance of adequate antimicrobial dosing as a determinant of outcome has been demonstrated in several randomized controlled trials performed in critically ill patients with nosocomial pneumonia [22-24]. Potentially, inadequately dosed antibiotics (ceftobiprole and tigecycline) were associated with statistically greater treatment failures and mortality compared to more optimally dosed comparators. Additionally, the results from two recent meta-analyses examining prolonged infusion of β-lactam antibiotics found that continuous infusion of β-lactam antibiotics led to the same clinical results as similar or higher dosed intermittent infusion antibiotic therapy [25,26]. However, in one of these meta-analyses, a trend towards benefit among patients receiving intermittent infusion antibiotics possibly explained by the use of higher antibiotic doses was observed [26]. The findings from a recent study examining epithelial lining fluid (ELF) concentrations of doripenem in normal volunteers reported the area under the curve ELF to plasma ratios was comparable to other carbapenems and supports further the use of the 1 g dose versus a 500 mg dose administered as a four-hour infusion to achieve higher doripenem levels in the ELF [27].

A population PK analysis to characterize the PK of doripenem in patients with VAP using data from subjects treated with doripenem from this study and patients with VAP from previous studies showed good PK coverage above MICs in this study, including the subjects with supranormal creatinine clearance. In addition, the population PK/PD analysis demonstrated no association between clinical outcomes and the infecting pathogen MICs within the PK dataset from this study. Plasma levels of doripenem were collected between study Days 2 and 3 so the ability to determine these doripenem exposure-response relationships closer to the time that the study endpoint was assessed was limited. However, notably, for subjects in both treatment arms, cure rates and mortality did not appear to increase with increasing MIC of the pathogens, suggesting conditions other than MIC and antibiotic dosing played a role in outcome. Furthermore, the mean distribution of CPIS values during antibiotic treatment for the first seven days of the study was similar for the two treatment groups (Figure 3), only separating after completion of therapy in the doripenem arm. This finding also suggests that the duration of antibiotic therapy and not the antibiotics themselves contributed to the observed differences in outcomes.

Another potential explanation for our findings is that achievement of the targeted antibiotic concentration goals took longer with the use of the prolonged infusion compared to the shorter infusions of the time-dependent carbapenems [28,29]. This could result in a delay in clearance of the infection that could adversely influence outcomes. The authors of a recent systematic review describing the pharmacokinetics of β-lactam antibiotics in the critically ill found that β-lactam antibiotic half-life and time above the MIC were virtually unpredictable, especially in those with normal renal function [30]. Moreover, two recent studies found that creatinine clearance appears to be an important predictor of sub-therapeutic β-lactam concentrations in critically ill patients [31,32]. Administration of a loading dose of doripenem might have improved concentration target attainment potentially allowing for a shorter course of effective therapy, even in the setting of increased drug clearance [30,32].

This study has several important limitations. First, clinical outcomes were assessed within 24 hours after administration of the last dose of blinded study drug. The timing of this visit provided three days for patients in the doripenem arm but less than 24 hours in the imipenem-cilastatin arm to relapse. Had the clinical outcome assessment been postponed to a few days later, additional relapses may have been observed in the imipenem-cilastatin arm. Second, premature closure of the study limited the number of patients in the MITT group and pathogen-specific subgroups. Third, there were a large number of study sites located in many countries which likely have different treatment practices, introducing additional variability. Fourth, enrollment occurred over the course of three years and we cannot exclude temporal changes in supportive care or other practices also having introduced additional variability. Fifth, allowing pathogens with MICs greater than 8 μg/mL may have influenced our results, especially for the shorter course of therapy. Sixth, there were numerically more cases of VAP attributed to *P. aeruginosa, A. baumannii *and MRSA in the doripenem arm compared to the imipenem-cilastatin arm and other imbalances in baseline characteristics between treatment groups which may have contributed to the study findings. Lastly, there may be additional imbalances between the two study groups that could have increased the severity of VAP (for example, corticosteroid therapy, chronic obstructive pulmonary disease) that were not included in our analysis.

## Conclusions

In summary, we demonstrated that a fixed 7-day course of doripenem was found to have non-significant higher rates of clinical failure and mortality compared to a fixed 10-day course of imipenem-cilastatin and a statistically greater mortality for the subgroup of VAP attributed to *P. aeruginosa*. Given the increasing prevalence of VAP caused by MDR Gram-negative bacteria, it is imperative that optimal antimicrobial treatment strategies be employed to optimize efficacy while minimizing the emergence of further antibiotic resistance [33]. In countries where doripenem 500 mg one-hour and four-hour infusions are approved to treat adults with nosocomial pneumonia, including VAP, the usual treatment duration is 7 to 14 days and should be guided by the severity of illness, infecting pathogen and the patients' clinical response with consideration given to treating patients with VAP for more than 7 days to optimize clinical outcome. Moreover, the European Medicines Agency (EMA) has recently recommended that doripenem 1 g doses administered every eight hours as four-hour infusions be considered in patients with augmented renal clearance (particularly those with creatinine clearance ≥150 ml/min) and/or in infections due to non-fermenting Gram-negative pathogens, such as *P. aeruginosa *and *Acinetobacter *spp. Moreover, the EMA highlighted that the usual treatment duration for patients with nosocomial pneumonia, including VAP, is 10 to 14 days and often in the upper range for patients infected with NLFGNB [34].

## Key messages

• A fixed 7-day course of doripenem was found to have non-significant higher rates of clinical failure and mortality compared to a fixed 10-day course of imipenem-cilastatin for the treatment of VAP.

• VAP due to NLFGNB should be treated with antibiotic courses that are longer than seven days.

• The use of prolonged infusion antibiotic therapy for VAP needs additional study to determine its relative efficacy compared to standard therapy.

## Abbreviations

AE: adverse event; APACHE: Acute Physiology and Chronic Health Evaluation; BAL: bronchoalveolar lavage; CI: confidence interval; CPIS:clinical pulmonary infection score; ELF: epithelial lining fluid; EMA: European Medicines Agency; EOT: end of therapy; ESBL: extended-spectrum beta-lactamase; FiO_2_: fraction of inspired oxygen; GCP: good clinical practices; ICU: intensive care unit; IDMC: Independent Data Monitoring Committee; ITT: intent-to-treat; MDR: multidrug-resistant; MIC: minimum inhibitory concentration; MITT: microbiological intent-to-treat; MRSA: methicillin-resistant *Staphylococcus aureus; *NLFGNB: non-lactose fermenting Gram-negative bacteria; PaO_2_: partial pressure of arterial oxygen; PD: pharmacodynamic; PK: pharmacokinetic; VAP: ventilator-associated pneumonia

## Competing interests

This study was funded by Janssen Pharmaceutical Research and Development. Dr. Kollef's effort was supported by the Barnes-Jewish Hospital Foundation and Dr. Kollef has received consulting fees from Janssen. Dr. Restrepo's time is partially protected by Award Number K23HL096054 from the National Heart, Lung, and Blood Institute. The content of this manuscript is solely the responsibility of the authors and does not necessarily represent the official views of the National Heart, Lung, and Blood Institute or the National Institutes of Health." The funding agencies had no role in the preparation, review or approval of the manuscript. The views expressed in this article are those of the authors and do not necessarily represent the views of the Department of Veterans Affairs, nor the University of Texas Health Science Center at San Antonio. The remaining authors have no competing interests to declare.

## Authors' contributions

MK, JC, MC, MR, MB, KK and RR had full access to all of the data in the study and take responsibility for the integrity of the data and the accuracy of the data analysis. MK, JC, MC, MR, MB, KK, RR, IC and HK contributed to the study conception and design, statistical analysis, drafting of the manuscript, and have given approval to the final version. IC and HK were the sponsor's designated clinical pharmacologist and modeling scientist responsible for pharmacokinetic analysis of the data and critical revision of the manuscript.

## Supplementary Material

Additional file 1 **A randomized trial of 7-day doripenem versus 10-day imipenem-cilastatin for ventilator-associated pneumonia on-line supplement**. Provides additional methods section, results and the IDMC charter. Click here for file
