# Supplementary material for: A randomized trial of 7-day doripenem versus 10-day imipenem-cilastatin for ventilator-associated pneumonia
Source: Crit Care. 2012 Nov 13;16(6):R218. doi: 10.1186/cc11862 (PMC3672596; doi:10.1186/cc11862)
Supplement: Additional file 1 — A randomized trial of 7-day doripenem versus 10-day imipenem-cilastatin for ventilator-associated pneumonia on-line supplement. Provides additional methods section, results and the IDMC charter. [file cc11862-S1.DOC]

**RANDOMIZED TRIAL OF 7-DAY DORIPENEM VERSUS 10-DAY IMIPENEM-CILASTATIN FOR VENTILATOR-ASSOCIATED PNEUMONIA**

Marin H. Kollef, MD^1^ [mkollef@dom.wustl.edu](mailto:mkollef@dom.wustl.edu), Jean Chastre, MD^2^ [Jean.Chastre@psl.aphp.fr](mailto:Jean.Chastre@psl.aphp.fr); Marc Clavel, MD^3^ [Marc.Clavel@chu-limoges.fr](mailto:Marc.Clavel@chu-limoges.fr); Marcos I. Restrepo, MD^4^ [restrepom@uthscsa.edu](mailto:restrepom@uthscsa.edu); Bart Michiels, PhD^5^ [BMichiel@its.jnj.com](mailto:BMichiel@its.jnj.com); Koné Kaniga, PhD^6^ [KKaniga@its.jnj.com](mailto:KKaniga@its.jnj.com); Iolanda Cirillo, MBS^6^ [ICirillo@its.jnj.com](mailto:ICirillo@its.jnj.com);

Holly Kimko, PhD^6^ [HKimko@its.jnj.com](mailto:HKimko@its.jnj.com); Rebecca Redman, MD^6^ [RRedman@its.jnj.com](mailto:RRedman@its.jnj.com)

^1^Division of Pulmonary and Critical Care Medicine, Washington University School of Medicine, St. Louis, Missouri; ^2^Institut de Cardiologie, Groupe Hospitalier Pitié-Salpêtrière, Paris, France; ^3^Centre Hospitalier De Limoges, Hospital Dupuytren, Limoges, France; ^4^Division Pulmonary/Critical Care Medicine, University of Texas Health Science Center at San Antonio and Division Pulmonary/Critical Care Medicine, South Texas Veterans Health Care System; ^5^Janssen Research and Development, Beerse, Belguim; ^6^Janssen Pharmaceutical Research and Development, Raritan, New Jersey

**Brief Description:** A prospective, double-blinded, randomized trial comparing a fixed 7-day course of doripenem 1 gram as a 4-hour infusion every 8 hours with a fixed 10-day course of imipenem-cilastatin 1 gram as a 1-hour infusion every 8 hours (April 2008 through June 2011). The clinical cure rate at the end of therapy (EOT) in the microbiological intent-to-treat (MITT) analysis was numerically lower for patients in the doripenem arm compared to the imipenem-cilastatin arm (45.6% versus 56.8%; 95% CI, -26.3% to 3.8%). All cause 28-day mortality in the MITT group was numerically greater for patients in the doripenem arm compared to the imipenem-cilastatin arm (21.5% versus 14.8%; 95% CI, -5.0 to 18.5) and for patients with *Pseudomonas aeruginosa* VAP (35.3% versus 0.0%; 95% CI, 12.6 to 58.0).

**Corresponding Author:**

Marin H. Kollef, MD

Division of Pulmonary and Critical Care Medicine

Washington University School of Medicine

660 South Euclid Avenue, Campus Box 8052

St. Louis, MO 63110

Phone (314) 454-8764; Fax (314) 454-5571

[mkollef@dom.wustl.edu](mailto:mkollef@dom.wustl.edu)

**ADDITIONAL FILE**

**Figure 3**

**Setting and Participants**

Patients were enrolled from 56 sites in 19 countries. Male and nonpregnant female patients aged ≥18 years were eligible for enrollment if they had pneumonia acquired after at least 48 hours of mechanical ventilation, had been hospitalized or been in a chronic care facility for a total of 5 or more days within the last 90 days, and had a baseline Clinical Pulmonary Infection Score (CPIS) > 6 and an Acute Physiology and Chronic Health Evaluation (APACHE) II score > 8 and < 35. Patients were required to have new or worsening radiographic infiltrates consistent with VAP and at least one of the following: fever (in the absence of fever-reducing agents) defined as a rectal temperature greater than 39^0^C or an increase in core temperature of greater than 1^0^C; hypothermia, defined as a rectal/core body temperature of less than 35^0^C; or white blood cell count >10,000 cells/mm^3^.

Patients were excluded if they had received antibiotics for the current episode of VAP for >24 hours before study drug was assigned, had known presence at baseline of only methicillin-resistant *Staphylococcus aureus* (MRSA) or *Stenotrophomonas maltophilia* infection, or acute respiratory distress syndrome. Patients were also excluded if they had any of the following conditions that could interfere with the assessment or interpretation of the diagnosis of VAP or response to therapy: chest trauma with severe lung bruising or loss of stability of the thoracic cage following a fracture of the sternum, ribs, or both, pleural effusion or empyema requiring drainage, lung cancer within the last 2 years, chronic bronchitis with an increase in disease severity within the last 30 days, chronic enlargement of the bronchi or bronchioles related to inflammatory disease or obstruction, lung abscess(s), anatomical bronchial obstruction, respiratory tuberculosis on treatment, suspected atypical pneumonia, chemical pneumonitis (e.g., aspiration of gastric contents, inhalation injury), cystic fibrosis, congestive heart failure, active seizure disorder within the last 2 years or brain injury such that imipenem-cilastatin would not be administered to the patient in usual practice, severe burns to greater than 15% of the body, evidence of severe and chronic liver disease indicating cirrhosis in the opinion of the investigator, and a history of hypersensitivity reactions to carbapenems, penicillins, other beta-lactam antibiotics, or beta-lactamase inhibitors.

A sample of fluid from each patient had to be obtained for Gram stain and culture by bronchoalveolar lavage (BAL) or mini-BAL prior to administration of study drug. Patients were started on empiric study drug therapy prior to the results of the culture being known. Patients whose culture results from the BAL/mini-BAL yielded at least one bacterial pathogen that grew at a density of ≥10^4^ CFU/mL and with an imipenem-cilastatin minimum inhibitory concentration (MIC) ≤ 8 μg/mL based on local MIC testing (or missing imipenem-cilastatin MIC) were allowed to continue receiving study drug therapy. Patients whose BAL/min-BAL culture results did not yield at least one bacterial pathogen meeting these criteria were to be discontinued from study drug therapy but were to be continued in the study and followed for safety. Pathogens isolated at the local laboratory were submitted to a central laboratory for confirmation of identity and susceptibility testing using the Clinical and Laboratory Standards Institute (CLSI) broth microdilution method M7-A7 (1).

Five blood samples were collected at 1, 2, 3, 4, and 6 hours from the start of infusion of the study drug between Days 2 and 3 from subjects enrolled at PK-participating sites.

**STATISTICAL MONITORING GUIDELINE INDEPENDENT DATA MONITORING COMMITTEE (IDMC)**

The primary purpose of the IDMC was to ensure the safety of the subjects in this study by identifying trends that may signify safety and efficacy concerns. Based on any trend(s) identified and the evaluation of the overall risk/benefit, the IDMC could recommend termination of this study due to safety concerns. The IDMC could also recommend termination of the study for lack of efficacy resulting in a treatment of subjects with a less efficacious drug. To address the above objectives, treatment-blinded safety and efficacy data from subjects in the present study (DORI-NOS3008) who received at least one dose or a partial dose of study drug therapy, as well as summarized safety data from the prior VAP registration trials (DORI-10 and the VAP portion of DORI-NOS2001) were provided by the Doripenem Independent Data Monitoring Committee Charter external statistician to the IDMC for the review meeting. The rationale for including safety data from DORI-10 and the VAP portion of DORI-NOS2001 was to aid the committee in identifying potential safety issues based on cumulative doripenem experience for similar indications. Note that the actual treatment labels for the DORI-NOS3008 data were masked as “Treatment A and Treatment B” to prevent inadvertent unblinding. The external statistician had access to the actual treatment codes and could reveal them to the IDMC should the need arise. The IDMC reviewed these data and made a recommendation as to whether the study should be continued. The objectives of the interim analyses from the IDMC charter are presented below:

**Safety Objective:**

1) To evaluate the overall safety of a 7-day course of doripenem 1 gram administered intravenously over 4 hours every 8 hours in subjects with VAP.

**Assessment of Safety Objective:**

1) To summarize safety data by adverse events, incidence of serious adverse events, study drug-related adverse events, study drug-related serious adverse events, discontinuation due to study drug-related adverse events, incidence of deaths and pre-specified laboratory test results (laboratory abnormalities). In addition to the summary of adverse events by preferred term, the following adverse events of special interest were to be summarized: anemia, hepatic injury, renal failure/impairment and seizures. Furthermore, other adverse events that have known association with parenteral carbapenem therapy were to be presented regardless of their

incidence. These adverse event terms were to be grouped together by high level group terms based on their relationship with each other.

2) To compare treatment groups using 2-sided confidence intervals (CIs). The 2-sided 80%, 90% and 95% CIs were to be calculated for the difference between treatments in the proportion of subjects with the following: serious adverse events, study drug-related adverse events, and incidence of deaths.

**Decision Rule:**

1) If the incidence rate of a preferred adverse event term was higher in one treatment arm than the other by more than 5%, then individual cases of the adverse event term would be reviewed to ascertain plausible relationship of the adverse event to study drug. Assuming sample sizes of 85 and 75 for doripenem and imipenem respectively, an overall true proportion of preferred adverse event terms equaling 5%, and there are *n* independent preferred adverse event terms, the probability that at least one preferred adverse event term has a difference of 5% between the 2 treatment arms is 1-(0.96)*n*. If *n*=10, the probability that at least one preferred term has 5% difference in treatment groups is 0.3. The probability increases with an increase in the number of preferred adverse event terms. In addition, the probability decreases with increases in the sample sizes of the treatment groups.

2) If the 90% CI for serious adverse events or study drug-related adverse events, or the

80% CI for the incidence of death does not include zero, the IDMC would request unblinding the treatment label and review individual cases to ascertain plausible relationship of these events to study drug.

**Efficacy Objective:**

1) To ensure that the 7-day course of doripenem treatment does not result in unacceptably higher failure rates compared to imipenem-cilastatin therapy in subjects with VAP caused by specific gram-negative pathogens (i.e., *Enterobacteriaceae, P. aeruginosa*, and *Acinetobacter*).

**Assessment of Efficacy Objective:**

1) To compare the treatment groups by calculating the observed difference in the percentage of subjects who are clinically cured at the end-of-therapy (EOT) visit and the corresponding 2-sided 90% CI for the treatment difference. The analysis was to be performed in the microbiologically modified intent-to-treat (mMITT) and the microbiologically evaluable (ME) analysis sets.

2) A similar comparison would be performed between the treatment groups in the microbiologically evaluable group at early follow-up (ME at EFU) for the subset of subjects with infections due to the gram-negative pathogens specified in the objective who were clinically cured at the EOT visit but relapse (failed) at the EFU visit.

3) In addition, a comparison would be performed between the treatment groups in the above analysis sets for all subjects regardless of the type of pathogen causing the infection.

**Decision Rules:**

The IDMC would request unblinding of the treatment labels if any of the

following occurred.

1) The 2-sided 90% CI for the between-group difference in clinical cure rate at the EOT visit among mMITT subjects with VAP caused by specific gram-negative pathogens (i.e., Enterobacteriaceae, *P. aeruginosa*, and *Acinetobacter*) did not include 0. If the true

imipenem-cilastatin cure rate in these subjects was 60% and the true doripenem cure rate was at least 60%, the futility criterion above could be translated to a difference of approximately 33% in the clinical cure rate between the treatment groups. Increases in the sample sizes of the treatment groups would lead to a smaller difference in the clinical cure rate between the groups.

2) The 2-sided 90% CI for the between-group difference in relapse rate at the EFU visit among ME at EFU subjects with VAP caused by specific gram-negative pathogens (i.e., Enterobacteriaceae, *P. aeruginosa*, and *Acinetobacter*) who were clinically cured at the EOT visit did not include 0. After treatment label was unblinded, if the cure rate at the EOT visit was lower in the doripenem arm compared to the imipenem-cilastatin arm and the 2-sided 90% CI did not include 0, or the relapse rate at the EFU visit was higher in the doripenem arm compared to the imipenem arm and the 2-sided 90% CI did not include 0, then the IDMC may recommend termination of the study.

**REFERENCES**

1. Clinical and Laboratory Standards Institute (CLSI). Methods for Dilution Antimicrobial Susceptibility Tests for Bacteria that Grow Aerobically; Approved Standard–7th ed. CLSI Document M7-A7. CLSI, 940 West Valley Rd., Suite 1400, Wayne, PA 19087. 2006.

| **Table S1: 28-Day Mortality of Baseline Pneumonia Pathogens by MIC of Treatment Received** **MITT Analysis Set** | | | | | | |
| --- | --- | --- | --- | --- | --- | --- |
|  | Doripenem | | | Imipenem | | |
|  | N | n | % | N | n | % |
| **Gram-negative, Aerobic** | | | | | | |
| *Acinetobacter baumannii* |  |  |  |  |  |  |
| Missing | 2 | 1 | 50 | 0 | 0 |  |
| 0.03 | 1 | 0 | 0 | 0 | 0 |  |
| 0.12 | 1 | 0 | 0 | 0 | 0 |  |
| 0.25 | 0 | 0 |  | 2 | 0 | 0 |
| 0.5 | 2 | 0 | 0 | 1 | 0 | 0 |
| 1 | 3 | 0 | 0 | 4 | 1 | 25 |
| 2 | 2 | 1 | 50 | 1 | 1 | 100 |
| 4 | 1 | 0 | 0 | 0 | 0 |  |
| 8 | 2 | 1 | 50 | 2 | 1 | 50 |
| 16 | 2 | 0 | 0 | 0 | 0 |  |
| 64 | 2 | 2 | 100 | 2 | 0 | 0 |
| 128 | 0 | 0 |  | 1 | 0 | 0 |
| >128 | 1 | 0 | 0 | 0 | 0 |  |
| *Acinetobacter* Species (Not Speciated) |  |  |  |  |  |  |
| 0.03 | 1 | 0 | 0 | 0 | 0 |  |
| *Burkholderia Gladioli* |  |  |  |  |  |  |
| 1 | 0 | 0 |  | 1 | 0 | 0 |
| *Chryseobacterium indologenes* |  |  |  |  |  |  |
| 4 | 0 | 0 |  | 1 | 0 | 0 |
| *Citrobacter freundii* |  |  |  |  |  |  |
| 0.12 | 0 | 0 |  | 2 | 1 | 50 |
| 0.5 | 0 | 0 |  | 1 | 1 | 100 |
| *Citrobacter koseri* |  |  |  |  |  |  |
| 0.06 | 1 | 0 | 0 | 0 | 0 |  |
| *Enterobacter aerogenes* |  |  |  |  |  |  |
| 0.25 | 0 | 0 |  | 1 | 0 | 0 |
| 0.5 | 0 | 0 |  | 2 | 1 | 50 |
| *Enterobacter asburiae* |  |  |  |  |  |  |
| 0.06 | 1 | 0 | 0 | 0 | 0 |  |
| *Enterobacter cloacae* |  |  |  |  |  |  |
| 0.03 | 5 | 1 | 20 | 0 | 0 |  |
| 0.06 | 2 | 0 | 0 | 0 | 0 |  |
| 0.12 | 0 | 0 |  | 1 | 0 | 0 |
| 0.25 | 1 | 1 | 100 | 2 | 0 | 0 |
| 0.5 | 0 | 0 |  | 1 | 0 | 0 |
| 1 | 0 | 0 |  | 1 | 0 | 0 |
| MITT = Microbiological Intent-to-Treat; MIC= minimum inhibitory concentration; N=number with MIC; n=number dying within 28 days; %= % dying within 28 days. | | | | | | |
| **Table S1 (Continued): 28-Day Mortality of Baseline Pneumonia Pathogens by MIC of Treatment Received** **MITT Analysis Set** | | | | | | |
|  | Doripenem | | | Imipenem | | |
|  | N | n | % | N | n | % |
| **Gram-negative, Aerobic** |  |  |  |  |  |  |
| *Enterobacter* Species (Not Speciated) |  |  |  |  |  |  |
| Missing | 1 | 0 | 0 | 0 | 0 |  |
| *Escherichia coli* |  |  |  |  |  |  |
| Missing | 0 | 0 |  | 1 | 0 | 0 |
| ≤ 0.015 | 4 | 0 | 0 | 0 | 0 |  |
| 0.03 | 2 | 1 | 50 | 0 | 0 |  |
| 0.06 | 0 | 0 |  | 3 | 0 | 0 |
| 0.12 | 0 | 0 |  | 8 | 1 | 12.5 |
| 0.25 | 0 | 0 |  | 2 | 1 | 50 |
| *Haemophilus influenzae* |  |  |  |  |  |  |
| Missing | 0 | 0 |  | 1 | 0 | 0 |
| ≤ 0.015 | 1 | 1 | 100 | 0 | 0 |  |
| 0.03 | 1 | 1 | 100 | 0 | 0 |  |
| 0.06 | 1 | 0 | 0 | 0 | 0 |  |
| 0.12 | 2 | 0 | 0 | 2 | 0 | 0 |
| 0.5 | 2 | 0 | 0 | 3 | 0 | 0 |
| 1 | 1 | 0 | 0 | 5 | 1 | 20 |
| 2 | 0 | 0 |  | 1 | 0 | 0 |
| 4 | 0 | 0 |  | 1 | 0 | 0 |
| *Haemophilus parainfluenzae* |  |  |  |  |  |  |
| Missing | 1 | 0 | 0 | 0 | 0 |  |
| 0.25 | 0 | 0 |  | 1 | 0 | 0 |
| *Haemophilus* Species (Not Speciated) |  |  |  |  |  |  |
| Missing | 1 | 0 | 0 | 0 | 0 |  |
| *Klebsiella oxytoca* |  |  |  |  |  |  |
| 0.06 | 1 | 0 | 0 | 0 | 0 |  |
| 0.12 | 0 | 0 |  | 1 | 0 | 0 |
| *Klebsiella pneumoniae* |  |  |  |  |  |  |
| Missing | 1 | 0 | 0 | 0 | 0 |  |
| ≤ 0.015 | 1 | 0 | 0 | 0 | 0 |  |
| 0.03 | 10 | 2 | 20 | 0 | 0 |  |
| 0.06 | 5 | 1 | 20 | 6 | 2 | 33.3 |
| 0.12 | 1 | 0 | 0 | 13 | 2 | 15.4 |
| 0.25 | 0 | 0 |  | 1 | 0 | 0 |
| 0.5 | 1 | 0 | 0 | 0 | 0 |  |
| *Moraxella catarrhalis* |  |  |  |  |  |  |
| Missing | 0 | 0 |  | 1 | 0 | 0 |
| 0.06 | 0 | 0 |  | 2 | 0 | 0 |
|  |  |  |  |  |  |  |
|  |  |  |  |  |  |  |
| **Table S1 (Continued): 28-Day Mortality of Baseline Pneumonia Pathogens by MIC of Treatment Received** **MITT Analysis Set** | | | | | | |
|  | Doripenem | | | Imipenem | | |
|  | N | n | % | N | n | % |
| **Gram-negative, Aerobic** |  |  |  |  |  |  |
| *Neisseria meningitides* |  |  |  |  |  |  |
| 0.06 | 1 | 0 | 0 | 0 | 0 |  |
| *Neisseria mucosa* |  |  |  |  |  |  |
| 0.25 | 0 | 0 |  | 1 | 0 | 0 |
| *Neisseria sicca* |  |  |  |  |  |  |
| 0.12 | 0 | 0 |  | 1 | 0 | 0 |
| *Neisseria* Species (Not Speciated) |  |  |  |  |  |  |
| Missing | 1 | 0 | 0 | 0 | 0 |  |
| *Pantoea agglomerans* |  |  |  |  |  |  |
| 0.03 | 1 | 0 | 0 | 0 | 0 |  |
| *Proteus mirabilis* |  |  |  |  |  |  |
| ≤ 0.015 | 1 | 0 | 0 | 0 | 0 |  |
| 0.06 | 1 | 0 | 0 | 0 | 0 |  |
| 0.25 | 1 | 0 | 0 | 0 | 0 |  |
| 0.5 | 1 | 1 | 100 | 1 | 0 | 0 |
| 1 | 0 | 0 |  | 1 | 0 | 0 |
| 2 | 0 | 0 |  | 3 | 1 | 33.3 |
| *Proteus vulgaris* |  |  |  |  |  |  |
| 0.5 | 1 | 0 | 0 | 0 | 0 |  |
| 2 | 0 | 0 |  | 1 | 0 | 0 |
| *Providencia* Species (Not Speciated) |  |  |  |  |  |  |
| Missing | 0 | 0 |  | 1 | 0 | 0 |
| *Pseudomonas aeruginosa* |  |  |  |  |  |  |
| Missing | 2 | 0 | 0 | 0 | 0 |  |
| 0.06 | 3 | 2 | 66.7 | 0 | 0 |  |
| *Pseudomonas aeruginosa* |  |  |  |  |  |  |
| 0.12 | 3 | 1 | 33.3 | 0 | 0 |  |
| 0.25 | 3 | 0 | 0 | 0 | 0 |  |
| 0.5 | 2 | 2 | 100 | 1 | 0 | 0 |
| 1 | 2 | 0 | 0 | 5 | 0 | 0 |
| 2 | 2 | 1 | 50 | 3 | 0 | 0 |
| 4 | 1 | 0 | 0 | 1 | 0 | 0 |
| 16 | 0 | 0 |  | 3 | 0 | 0 |
| 32 | 0 | 0 |  | 3 | 0 | 0 |
| 64 | 2 | 1 | 50 | 0 | 0 |  |
| 128 | 2 | 0 | 0 | 1 | 0 | 0 |
| >128 | 0 | 0 |  | 1 | 0 | 0 |
| *Pseudomonas stutzeri* |  |  |  |  |  |  |
| 0.03 | 1 | 0 | 0 | 0 | 0 |  |
|  | | | | | | |
| **Table S1 (Continued): 28-Day Mortality of Baseline Pneumonia Pathogens by MIC of Treatment Received** **MITT Analysis Set** | | | | | | |
|  | Doripenem | | | Imipenem | | |
|  | N | n | % | N | n | % |
| **Gram-negative, Aerobic** |  |  |  |  |  |  |
| *Serratia marcescens* |  |  |  |  |  |  |
| 0.03 | 1 | 0 | 0 | 0 | 0 |  |
| 0.12 | 2 | 0 | 0 | 0 | 0 |  |
| 0.25 | 1 | 0 | 0 | 2 | 0 | 0 |
| 0.5 | 0 | 0 |  | 1 | 0 | 0 |
| 1 | 0 | 0 |  | 2 | 1 | 50 |
| *Stenotrophomonas maltophilia* |  |  |  |  |  |  |
| Missing | 0 | 0 |  | 1 | 1 | 100 |
| 64 | 2 | 0 | 0 | 0 | 0 |  |
| 128 | 1 | 0 | 0 | 1 | 0 | 0 |
| >128 | 1 | 0 | 0 | 1 | 0 | 0 |
| **Gram-positive, Aerobic** | | | | | | |
| *Corynebacterium amycolatum* |  |  |  |  |  |  |
| ≤ 0.015 | 0 | 0 |  | 1 | 0 | 0 |
| *Corynebacterium* Group G |  |  |  |  |  |  |
| ≤ 0.015 | 1 | 0 | 0 | 0 | 0 |  |
| *Corynebacterium* Species (Not Speciated) |  |  |  |  |  |  |
| Missing | 3 | 0 | 0 | 0 | 0 |  |
| *Corynebacterium striatum* |  |  |  |  |  |  |
| 0.5 | 1 | 1 | 100 | 0 | 0 |  |
| *Enterococcus faecalis* |  |  |  |  |  |  |
| 1 | 0 | 0 |  | 1 | 0 | 0 |
| 2 | 1 | 0 | 0 | 0 | 0 |  |
| 4 | 1 | 0 | 0 | 0 | 0 |  |
| *Staphylococcus aureus* |  |  |  |  |  |  |
| Missing | 1 | 0 | 0 | 2 | 0 | 0 |
| ≤ 0.015 | 12 | 1 | 8.3 | 21 | 3 | 14.3 |
| 0.03 | 4 | 1 | 25 | 1 | 0 | 0 |
| 0.5 | 2 | 1 | 50 | 0 | 0 |  |
| 2 | 2 | 1 | 50 | 0 | 0 |  |
| 8 | 1 | 0 | 0 | 0 | 0 |  |
| 16 | 2 | 1 | 50 | 0 | 0 |  |
| 64 | 0 | 0 |  | 1 | 1 | 100 |
| 128 | 0 | 0 |  | 1 | 0 | 0 |
| >128 | 0 | 0 |  | 1 | 0 | 0 |
| *Staphylococcus epidermidis* |  |  |  |  |  |  |
| 16 | 0 | 0 |  | 1 | 0 | 0 |
|  |  |  |  |  |  |  |
| **Table S1 (Continued): 28-Day Mortality of Baseline Pneumonia Pathogens by MIC of Treatment Received** **MITT Analysis Set** | | | | | | |
|  | Doripenem | | | Imipenem | | |
|  | N | n | % | N | n | % |
| *Streptococcus agalactiae* |  |  |  |  |  |  |
| Missing | 1 | 0 | 0 | 0 | 0 |  |
| ≤ 0.015 | 1 | 0 | 0 | 0 | 0 |  |
| **Gram-positive, Aerobic** | | | | | | |
| *Streptococcus anginosus* |  |  |  |  |  |  |
| ≤ 0.015 | 1 | 0 | 0 | 1 | 0 | 0 |
| *Streptococcus constellatus* |  |  |  |  |  |  |
| ≤ 0.015 | 0 | 0 |  | 1 | 0 | 0 |
| *Streptococcus pneumoniae* |  |  |  |  |  |  |
| ≤ 0.015 | 2 | 0 | 0 | 1 | 0 | 0 |
| 0.03 | 0 | 0 |  | 1 | 0 | 0 |
| 0.06 | 0 | 0 |  | 1 | 0 | 0 |
| 0.12 | 0 | 0 |  | 1 | 0 | 0 |
| 0.25 | 1 | 0 | 0 | 2 | 0 | 0 |
| 0.5 | 0 | 0 |  | 2 | 0 | 0 |
| *Streptococcus* Species (Not Speciated) |  |  |  |  |  |  |
| Missing | 1 | 0 | 0 | 1 | 0 | 0 |

| Table S2: Adverse Events Reported in at Least 3% of Subjects in Either Treatment Group by Organ Class and Preferred Term | | | |
| --- | --- | --- | --- |
|  | Doripenem | Imipenem | Total |
| **Body System Or Organ Class** | (N=115) | (N=112) | (N=227) |
|  | n (%) | n (%) | n (%) |
| **Total no. subjects with adverse events** | 106 (92.2) | 107 (95.5) | 213 (93.8) |
|  |  |  |  |
| **Blood and Lymphatic System Disorders** | 30 (26.1) | 30 (26.8) | 60 (26.4) |
| Anemia | 25 (21.7) | 25 (22.3) | 50 (22.0) |
| Leukocytosis | 4 ( 3.5) | 4 ( 3.6) | 8 ( 3.5) |
| Thrombocytopenia | 4 ( 3.5) | 5 ( 4.5) | 9 ( 4.0) |
|  |  |  |  |
| **Cardiac Disorders** | 24 (20.9) | 30 (26.8) | 54 (23.8) |
| Atrial Fibrillation | 10 ( 8.7) | 8 ( 7.1) | 18 ( 7.9) |
| Bradycardia | 3 ( 2.6) | 7 ( 6.3) | 10 ( 4.4) |
| Cardiac Arrest | 3 ( 2.6) | 6 ( 5.4) | 9 ( 4.0) |
| Tachycardia | 3 ( 2.6) | 5 ( 4.5) | 8 ( 3.5) |
|  |  |  |  |
| **Gastrointestinal Disorders** | 41 (35.7) | 43 (38.4) | 84 (37.0) |
| Constipation | 9 ( 7.8) | 10 ( 8.9) | 19 ( 8.4) |
| Diarrhea | 11 ( 9.6) | 13 (11.6) | 24 (10.6) |
| Impaired Gastric Emptying | 2 ( 1.7) | 5 ( 4.5) | 7 ( 3.1) |
| Nausea | 5 ( 4.3) | 7 ( 6.3) | 12 ( 5.3) |
| Vomiting | 6 ( 5.2) | 6 ( 5.4) | 12 ( 5.3) |
|  |  |  |  |
| **General Disorders and Administration Site Conditions** | 20 (17.4) | 23 (20.5) | 43 (18.9) |
| Hypothermia | 3 ( 2.6) | 4 ( 3.6) | 7 ( 3.1) |
| Peripheral Edema | 1 ( 0.9) | 5 ( 4.5) | 6 ( 2.6) |
| Pyrexia | 10 ( 8.7) | 10 ( 8.9) | 20 ( 8.8) |
|  |  |  |  |
| **Hepatobiliary Disorders** | 5 ( 4.3) | 4 ( 3.6) | 9 ( 4.0) |
| Cholestasis | 4 ( 3.5) | 2 ( 1.8) | 6 ( 2.6) |
|  |  |  |  |
| **Infections and Infestations** | 56 (48.7) | 58 (51.8) | 114 (50.2) |
| Bacteremia | 3 ( 2.6) | 5 ( 4.5) | 8 ( 3.5) |
| Device Related Infection | 1 ( 0.9) | 5 ( 4.5) | 6 ( 2.6) |
| Pneumonia | 10 ( 8.7) | 12 (10.7) | 22 ( 9.7) |
| Sepsis | 7 ( 6.1) | 4 ( 3.6) | 11 ( 4.8) |
| Septic Shock | 6 ( 5.2) | 6 ( 5.4) | 12 ( 5.3) |
| Urinary Tract Infection | 15 (13.0) | 16 (14.3) | 31 (13.7) |
|  |  |  |  |
| **Metabolism and Nutrition Disorders** | 29 (25.2) | 33 (29.5) | 62 (27.3) |
| Hyperglycemia | 3 ( 2.6) | 4 ( 3.6) | 7 ( 3.1) |
| Hyperkalemia | 5 ( 4.3) | 6 ( 5.4) | 11 ( 4.8) |
| Hypernatremia | 4 ( 3.5) | 7 ( 6.3) | 11 ( 4.8) |
| Hypocalcemia | 4 ( 3.5) | 3 ( 2.7) | 7 ( 3.1) |
| Hypoglycemia | 2 ( 1.7) | 4 ( 3.6) | 6 ( 2.6) |
| Hypokalemia | 12 (10.4) | 12 (10.7) | 24 (10.6) |
| Hypomagnesemia | 4 ( 3.5) | 7 ( 6.3) | 11 ( 4.8) |
| Hyponatremia | 5 ( 4.3) | 10 ( 8.9) | 15 ( 6.6) |
| Hyponatremia | 5 ( 4.3) | 10 ( 8.9) | 15 ( 6.6) |
|  |  |  |  |
| **Nervous System Disorders** | 14 (12.2) | 19 (17.0) | 33 (14.5) |
| Brain Edema | 3 ( 2.6) | 4 ( 3.6) | 7 ( 3.1) |
|  |  |  |  |
| **Psychiatric Disorders** | 32 (27.8) | 31 (27.7) | 63 (27.8) |
| Agitation | 11 ( 9.6) | 7 ( 6.3) | 18 ( 7.9) |
| Anxiety | 2 ( 1.7) | 6 ( 5.4) | 8 ( 3.5) |
| Confusional State | 3 ( 2.6) | 5 ( 4.5) | 8 ( 3.5) |
| Depression | 14 (12.2) | 7 ( 6.3) | 21 ( 9.3) |
| Insomnia | 8 ( 7.0) | 8 ( 7.1) | 16 ( 7.0) |
|  |  |  |  |
|  |  |  |  |
|  |  |  |  |
|  |  |  |  |
| **Table S2 (Continued): Adverse Events Reported in at Least 3% of Subjects in Either Treatment Group by Organ Class and Preferred Term** | | | |
| **Renal and Urinary Disorders** | 18 (15.7) | 20 (17.9) | 38 (16.7) |
| Hematuria | 2 ( 1.7) | 4 ( 3.6) | 6 ( 2.6) |
| Renal Failure | 5 ( 4.3) | 2 ( 1.8) | 7 ( 3.1) |
| Renal Failure Acute | 3 ( 2.6) | 6 ( 5.4) | 9 ( 4.0) |
| Renal Impairment | 5 ( 4.3) | 4 ( 3.6) | 9 ( 4.0) |
|  |  |  |  |
| **Respiratory, Thoracic and Mediastinal Disorders** | 40 (34.8) | 41 (36.6) | 81 (35.7) |
| Bronchospasm | 4 ( 3.5) | 6 ( 5.4) | 10 ( 4.4) |
| Dyspnea | 3 ( 2.6) | 5 ( 4.5) | 8 ( 3.5) |
| Hydrothorax | 5 ( 4.3) | 4 ( 3.6) | 9 ( 4.0) |
| Hypoxia | 3 ( 2.6) | 5 ( 4.5) | 8 ( 3.5) |
| Pleural Effusion | 5 ( 4.3) | 4 ( 3.6) | 9 ( 4.0) |
| Rhonchi | 1 ( 0.9) | 4 ( 3.6) | 5 ( 2.2) |
|  |  |  |  |
| **Skin and Subcutaneous Tissue Disorders** | 25 (21.7) | 23 (20.5) | 48 (21.1) |
| Decubitus Ulcer | 14 (12.2) | 11 ( 9.8) | 25 (11.0) |
|  |  |  |  |
| **Vascular Disorders** | 21 (18.3) | 25 (22.3) | 46 (20.3) |
| Hypertension | 2 ( 1.7) | 5 ( 4.5) | 7 ( 3.1) |
| Hypotension | 11 ( 9.6) | 10 ( 8.9) | 21 ( 9.3) |

**FIGURE LEGENDS**

**Figure S1.** Kaplan-Meier curves for probability of survival for the intention-to-treat

(ITT) population. (P=0.118, Log-Rank Test)

**eFigure 2.** Kaplan-Meier curves for probability of survival for the microbiological ITT

population. (P=0.260, Log-Rank Test)

**Figure S1**

**Figure S2**
